# The Proceedings of the Physicians’ Meeting

**Published:** 1858-04

**Authors:** 


*The Proceedings of the Phpsicians' Meeting.*—Pursuant to call for a meeting of the Medical profession of Cleveland and vicinity, to be held at the office of Doctors Ackley & Garlick, a very large number of physicians were in attendance.

A. S. Palmer, M. D., was called to the Chair, and John Holland was appointed Secretary.

On motion of the Chair, Dr. Garlick was called upon to explain the object of the meeting.

He stated briefly that the meeting was called to take into consideration the late action of the Faculty of the Cleveland Medical College, in recommending Dr. I. J. Tod as a person eminently qualified as Physician and Surgeon, to take charge of the Marine Hospital of this city.

Remarks were made by Doctors Ackley, Thrall, Strong, Sterling, Garlick, Gordon, and several others, it having been conclusively shown that Dr. Tod had not been practising medicine for the past thirty years, and it being equally evident that the faculty were aware of this when they signed the paper.

On motion of Dr. Capener, the Chair appointed Drs. Sterling, Garlick, and Gordon, a Committee to draft resolutions expressive of the sense of the meeting.

The Committee having retired for that purpose, remarks were made by Drs. Everett, Thrall, and other Physicians.

The Committee returned, and reported the preamble and resolutions, which were ordered to be read; and after considerable discussion, Dr. Nelson proposed their adoption.

The motion having been put by the Chair, the preamble and resolutions were adopted without a dissenting voice.

Whereas, It has come to the knowledge of this meeting, that the Faculty of the Cleveland Medical College did basely use their influence, and abuse the confidence and trust usually reposed in men occupying their position, by signing a certificate, directed to the Hon. Howell Cobb, wherein they knowingly, and in the opinion of this meeting, for sinister purposes, misrepresent the facts in the following words, to wit:

"Cleveland, February 1, 1858.

Sir.—It having come to our knowledge that efforts are being made by contemptible parties to request the removal of Dr. Tod, the present Physician of the Marine Hospital, we, as Professors of the Cleveland Medical College, take this opportunity of assuring you that we are intimately acquainted with Dr. I. J. Tod, and know him to be eminently qualified, both professionally and otherwise, to discharge the duties imposed by his position. And we can further assure you, that as long as he holds the post, the interests of the Government will be well cared for.

To the Hon. Howell Cobb, Secretary of U. S. Treasury."

*Resolved*, That the fact that Dr. Tod did abandon the practice of medicine as long ago as 1828, is beyond all doubt. That of this the Faculty were well advised; and that the said Dr. Tod has not, in any sense, been engaged as a practitioner of medicine since that time, until he assumed the duties of Physician and Surgeon of the United States Marine Hospital in this city.

*Resolved*, Therefore, that it is a self-evident fact, that Dr. I. J. Tod is eminently *unfit* to take charge of a Government Hospital, and that this the Faculty must have known when they signed the above certificate.

*Resolved*, That by this act, the Faculty of the Cleveland Medical College have forfeited all claims to the respect and patronage of the Profession.

*Resolved*, That the attempt of the Dean of the Faculty to justify this gross abuse of their position, when called upon by one of the Profession, by stating that when any particular skill was required at the Hospital, Prof. Webster (one of the Professors,) would attend to the case, is, at the best, but a miserable subterfuge, and a virtual acknowledgment that they know their statements to be untrue.

*Resolved*, That the assertion of one of the Faculty thnt Dr. Tod's fitness for that post was as good as that of Physicians in general of *this country*, is a base libel upon American Physicians—an insult to the Profession, and merits their indignation.

*Resolved*, That a Committee of three persons be appointed by the Chairman of this meeting, to lay this act of the Faculty before the Board of Medical Censors, at the approaching commencement of the College.

*Resolved*, That the medicals journals of our state, the Buffalo Medical Journal, and our city papers, be each furnished with a copy of the proceedings of this meeting, and be requested to publish the same in their respective journals and papers.

| | |
|---|---|
| John Holland, Secretary. | A. S. Palmer, M. D., President. |
| B. Sterling, M. D., | Wm. H. Capener, M. D., |
| P. A. Gordon, M. D, | N. Nelson, M. D., |
| T. Garlick, M. D., | J. Hammond, M. D., |
| Robt. S. Strong, M. D., | W. Dolan, M. D., |
| F. G. Thrall, M. D., | W. B. Rezner, M. D., |
| H. A. Ackley, M. D., | P. R. Everett, M. D., |
| A. Everett, M. D., | J. H. Marshall, M. D., |
| John J. Conlan, M. D., | Thos. S. Rodman, M. D., |

E. H. Sprague, M. D.

